# Measuring scientific creative thinking: development and validation of a process-oriented instrument

**DOI:** 10.3389/fpsyg.2026.1762573

**Published:** 2026-03-03

**Authors:** Huanhuan Lu, Shuaishuai Mi, Hualin Bi

**Affiliations:** 1 School of Education, Shandong Women’s University, Jinan, Shandong, China; 2 School of Physics and Electronics, Shandong Normal University, Jinan, Shandong, China; 3 College of Chemistry, Engineering and Materials Science, Shandong Normal University, Jinan, Shandong, China

**Keywords:** cognitive process, instrument development, process-oriented assessment, psychometrics, scientific creative thinking

## Abstract

Scientific creative thinking (SCT) is a critical cognitive process that drives scientific discovery and innovation. To address the limitations of existing assessment tools in capturing its multi-stage, domain-specific nature, this study developed and validated the Scientific Creative Thinking Scale (SCTS). Grounded in an integrative psychological framework, the SCTS combines explicit indicators of divergent thinking with implicit theories of within-domain appropriateness. The scale employs three open-ended tasks based on real-world scientific scenarios to systematically assess the three core stages of SCT: problem identification, hypothesis construction, and experimental verification. Using a two-stage independent sample design with 401 tenth-grade students, the scale was rigorously validated. Exploratory factor analysis (*n* = 182) employing Principal Axis Factoring (PAF) with parallel analysis extracted a stable three-factor structure, accounting for 70.189% of the total variance. Confirmatory factor analysis with an independent sample (*n* = 219) confirmed the three-factor model with good fit indices (CFI = 0.948, TLI = 0.922, RMSEA = 0.076, SRMR = 0.032). The scale demonstrated robust reliability: internal consistency was satisfactory (Cronbach’s *α* = 0.87; McDonald’s *ω* = 0.89), and inter-rater reliability was excellent (ICC ≥ 0.95). Composite reliability for each dimension exceeded 0.86, and average variance extracted exceeded 0.67, indicating good convergent validity. The Fornell-Larcker criterion was met, as the square root of AVE for each dimension exceeded its correlations with other factors, supporting discriminant validity. Additionally, the SCTS total score showed a significant positive correlation with science academic achievement (*ρ* = 0.55, *p* < 0.001), providing preliminary evidence for criterion-related validity. This study offers a process-oriented, psychometrically sound instrument for measuring SCT, laying a methodological foundation for future research on its cognitive mechanisms, individual differences, and developmental trajectories.

## 1 Introduction

Contemporary human society is confronted with a series of complex challenges, including climate change, public health crises, artificial intelligence ethics, and energy sustainability. These challenges are often characterized by their cross-disciplinary boundaries, uncertainty, and intricate structures (Clark and Wallace, 2015; Steiner, 2025). Addressing such problems requires individuals not only to integrate information and perspectives from diverse disciplines but also to propose solutions that transcend conventional approaches, and ultimately to translate these ideas into concrete, actionable plans. The core of Scientific Creative Thinking (SCT) lies precisely in generating high-quality, original, and elegant solutions to such complex, novel, and ill-defined scientific problems (Mumford et al., 2012; Ayas and Sak, 2014). Therefore, SCT not only serves as the core cognitive foundation of individual scientific creativity but also constitutes a fundamental competence for addressing global societal challenges, driving technological progress, and fostering sustainable social development (Steiner et al., 2015; Saleh and Brem, 2023).

From a cognitive psychology perspective, scientific creativity is far from a vague, holistic concept; it is a decomposable, multi-stage cognitive process. A central methodological challenge, however, lies in externalizing and objectively measuring this complex internal mental activity. The lack of precise measurement tools constrains both the assessment of individual differences in scientific creative potential and deeper investigation into the fundamental question of how creativity emerges. Therefore, developing a theoretically grounded, process-oriented assessment tool with sound psychometric properties is not merely an applied need, but foundational work essential for advancing cognitive theories of creativity and bridging the gap between neural mechanisms and observable behavior.

Despite significant advances in creativity research (Beghetto and Kaufman, 2010; Treffinger et al., 2001), measurement tools specifically designed for SCT remain relatively scarce. Existing instruments exhibit two primary limitations. First, regarding process completeness, most tools fail to systematically cover the core cognitive stages of scientific creative activity. Domain-general instruments, such as the Torrance Tests of Creative Thinking (TTCT) (Torrance, 1974), possess sound psychometric properties. However, their abstract tasks are detached from authentic scientific inquiry practices and thus struggle to capture the distinctive thinking characteristics inherent to the scientific domain, raising concerns about their ecological and construct validity (Zeng et al., 2011). While domain-specific tests represent an improvement, they still exhibit limitations. Early instruments, such as the tasks designed by Hu and Adey (2002) (e.g., “unusual uses,” “product improvement”), did not adequately simulate the authentic research processes through which scientists systematically explore natural phenomena. Subsequent instruments have deepened aspects such as hypothesis generation and experimental design (Sak and Ayas, 2013; Atesgoz and Sak, 2021; Yurt, 2025) and have introduced advances in assessment techniques (Weng et al., 2025). However, they have generally overlooked the critical front-end stage of problem identification (Einstein and Infeld, 1938). Research indicates that problem-finding ability may be even more strongly correlated with creativity than problem-solving; problem finding is not only a key component of the creative process but can also be reliably assessed (Chand and Runco, 1993). An ideal assessment instrument should encompass all critical stages of the creative process (Zeng et al., 2011).

Second, regarding assessment dimensions, existing instruments inadequately capture the core creativity element of appropriateness (Sak and Ayas, 2013; Atesgoz and Sak, 2021; Yurt, 2025; Weng et al., 2025). The scoring systems of these tools either treat appropriateness merely as a preliminary screening threshold for response plausibility or as a localized feature of a specific stage within SCT. They fail to recognize appropriateness as a core dimension that permeates and profoundly influences the entire scientific creative process. However, within the scientific context, “appropriateness” carries a richer meaning extending beyond general “plausibility.” It encompasses the research value of the problem, the testability of the hypothesis, the fundamental fit of the explanation, and the sophistication of the method. Judgments on these elements are highly dependent on the implicit norms and knowledge systems embedded within the scientific community.

To address these research gaps, this study aims to develop and validate the SCTS grounded in an explicit process model, from the perspectives of cognitive psychology and psychometrics. Compared to existing instruments, the SCTS offers three main improvements. First, framed around the complete scientific creative process, it formally incorporates problem identification as a core assessment component. Second, it elevates appropriateness to a core dimension parallel to novelty, operationalizing it based on the specific scientific creative process. Third, it enhances ecological validity by using authentic scientific scenarios as a thread, constructing task prototypes around fundamental scientific principles that require participants to engage in coherent creative thinking activities. In summary, the SCTS aims to provide an assessment approach for measuring SCT that offers greater process completeness and contextual relevance.

Based on this, the central research question this study seeks to answer is: What empirical evidence exists for the reliability and validity of the developed SCTS?

## 2 Theoretical foundation

This study is grounded in the established scholarly consensus that defines “creativity” as the ability to produce outcomes that are both novel and appropriate (Sternberg and Lubart, 1996; Piffer, 2012; Plucker et al., 2004). Scientific creativity, specifically, refers to the capacity to generate novel ideas or products that are relevant and scientifically useful within a given context (Ayas and Sak, 2014). Building on this foundation, the present study operationally defines SCT as the structured cognitive process of exploring and understanding the natural world, aimed at generating solutions to problems that are both novel (unique, unconventional) and appropriate (consistent with scientific norms, logically rigorous, and testable). The construction of the assessment framework is informed by the following core theoretical pillars.

### 2.1 Domain specificity and the hierarchical nature of creativity

Research indicates that the manifestation and assessment of creativity are significantly domain-dependent (Baer, 1998; Silvia et al., 2009). This specificity is primarily evidenced in three ways. First, creative thinking is highly reliant on a domain-specific knowledge base. The organization, accessibility, and fluent application of this knowledge profoundly influence the generation and quality of creative outputs (Alexander, 1992; Feldhusen, 2006; Mayer, 2006). Second, creative activities in different domains systematically demand distinct psychological characteristics and dominant cognitive modes. For instance, scientific creativity emphasizes logical reasoning and empirical verification, whereas artistic creativity relies more on esthetic perception and emotional expression (Kind and Kind, 2007); this difference is also reflected in the personality traits of creative individuals (Feldhusen, 2006). Third, cognitive neuroscience provides mechanistic evidence for domain specificity. For example, while scientific and artistic creativity share some foundational neural networks, they specifically activate brain regions associated with logical reasoning and problem-driven processing (e.g., cingulate gyrus) and those related to novelty-seeking and perceptual information processing (e.g., temporal lobe), respectively (Shen et al., 2010). Therefore, effective measurement of scientific creativity must be grounded in its unique domain-specific norms, knowledge systems, and cognitive logic.

Simultaneously, creative performance exists at different levels of maturity and recognition. Kaufman and Beghetto (2009) Four C model provides a key framework here, notably by articulating the concept of “mini-c” creativity—the novel and personally meaningful interpretations constructed by individuals during learning and experience. The SCTS developed in this study is designed to measure precisely this developmental cognitive process, corresponding to the “mini-c” level as exhibited during scientific problem-solving. This focus ensures the tool’s sensitivity to intrinsic, nascent creative thinking activities rather than solely to mature, publicly acknowledged creative products. Accordingly, SCTS tasks are not designed to simulate high-difficulty scientific discovery but use authentic, relatable scenarios to elicit learners’ scientific inquiry, thereby tapping into the personalized creative potential within their thought processes.

### 2.2 A three-stage cognitive process model of scientific creative thinking

Scientific creativity is fundamentally a structured, domain-specific form of complex problem-solving. It involves systematic cognitive exploration within a defined “problem space,” employing various strategies to transform an initial problem state into a desired goal state (Simon, 1977). Synthesizing analyses of scientific discovery (Mansfield and Busse, 1981; Klahr and Dunbar, 1988; Bliersbach and Reiners, 2017), this study proposes a three-stage SCT process model, deconstructing the continuous flow of creative thought into observable and assessable cognitive units:

- Problem Identification. This stage requires the thinker to detect anomalies, contradictions, gaps in knowledge, or potential, underexplored research value within complex natural or experimental phenomena with high sensitivity and insight. The core cognitive task is to precisely refine and define these vague perceptions or initial curiosities into clear, specific, and investigable scientific questions. This stage reflects cognitive sensitivity and insightfulness.
·Hypothesis Construction. Based on the identified problem, the thinker needs to mobilize their existing knowledge base, scientific theoretical frameworks, intuitive hunches, or cross-domain analogies to generate one or more speculative explanations or solutions—that is, scientific hypotheses—that possess explanatory power and are, in principle, testable. This stage embodies the generative, associative, and explanatory aspects of thinking.
- Experimental Verification. The thinker designs and rigorously implements corresponding empirical testing plans. This typically involves carefully constructed experiments but can also extend to systematic observation, computer simulation, and other scientific methods. This stage emphasizes the critical, logical, and empirically-oriented nature of thinking.

### 2.3 Implicit theories of creativity

To construct a comprehensive framework for assessing SCT, particularly to capture its indispensable “appropriateness” dimension, this study introduces implicit theories of creativity as a core theoretical basis. This theory posits that within a specific domain (e.g., science), knowledgeable practitioners (e.g., scientists or science teachers) can make stable and valid judgments about the quality of creative products based on their internalized, shared standards and consensus regarding “what constitutes good creative work in that domain” (Sternberg, 1985). This provides the methodological foundation for this study’s assessment of “appropriateness.” Based on this theory, the SCTS establishes “appropriateness” as a core, judgeable construct that permeates all stages of SCT. In the scientific context, its connotation is specifically manifested as research value, essence, testability, and effectiveness. This ensures the assessment attends not only to the novelty and quantity of ideas but also to their conformity with the internal logic and norms of scientific inquiry, thereby enhancing the domain-specific explanatory power and ecological validity of the measurement tool.

## 3 Conceptual framework

**Figure 1:**
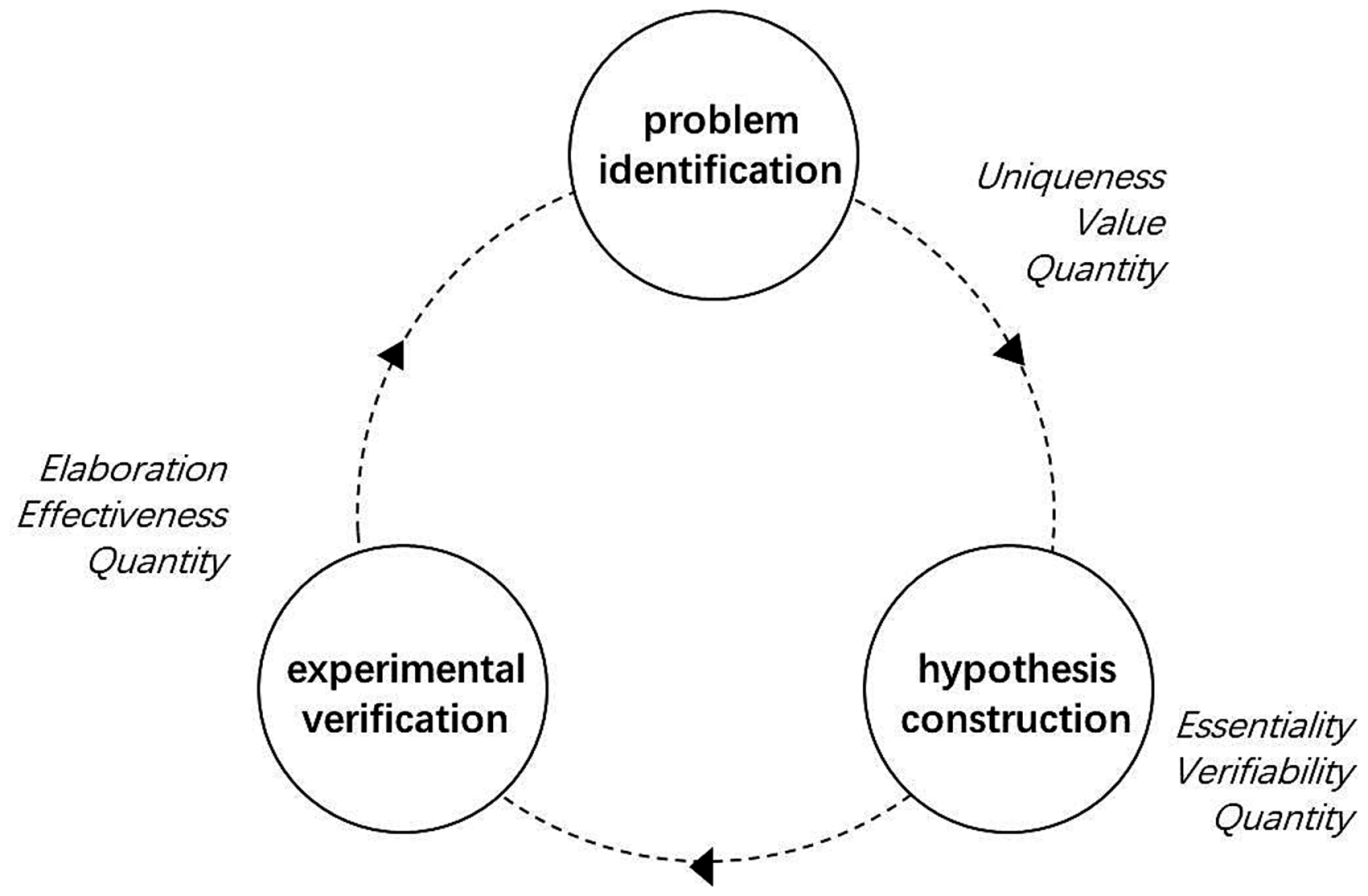
Conceptual framework of scientific creative thinking.

Based on the three-stage cognitive process model of SCT, this study constructed an integrative conceptual framework (Figure 1). This framework aims to systematically elucidate the core constitutive dimensions of SCT and the principles underlying its assessment, integrating explicit indicators of divergent thinking with implicit criteria of scientific appropriateness. Specifically, the divergent thinking indicators, derived from the classic theory of Guilford and others, are used to quantify the generative characteristics of thinking outputs (such as fluency and flexibility). The scientific appropriateness criteria, grounded in domain-specific implicit theories, are applied through qualitative judgments by knowledgeable raters to assess the fitness of thinking products with scientific logic and empirical norms.

Concretely, the framework is organized around the following three stages and their core dimensions:

- In the Problem Identification stage, the core dimensions include: (1) Uniqueness, which refers to proposing questions with a frequency of occurrence below 10% among all participants (referring to the definition by Hu and Han, 2006). Due to differences in the phenomenological features and scientific principles underlying the three task scenarios, the questions posed by students are context-specific, therefore, uniqueness is calculated separately for each scenario and is not aggregated across scenarios; (2) Value, judged by raters based on implicit theory, focusing on whether the question is purposefully clear, conducive to critical inquiry, and touches the essence of the phenomenon, serving as the core indicator of appropriateness for this stage; and (3) Quantity of Questions, directly reflecting the fluency of thinking.
- In the Hypothesis Construction stage, the core dimensions include: (1) Essentiality, meaning whether the hypothesis aims to explain the core internal mechanism of the problem, judged by raters as a key manifestation of appropriateness in this stage; (2) Verifiability, referring to whether the hypothesis can, in principle, be tested through experiment or observation, also a key dimension of appropriateness; and (3) Quantity of Hypotheses, reflecting the flexibility of thinking.
- In the Experimental Verification stage, the core dimensions include: (1) Effectiveness, meaning whether the designed plan can logically and rigorously test the target hypothesis, judged by raters as constituting the core of appropriateness for this stage; (2) Elaboration, assessing whether the plan is clear, specific, operational, and contains well-defined variables and control steps; and (3) Quantity of Verification Methods, used to measure the fluency of solution generation.

## 4 Research method

### 4.1 Instrument development

Based on the conceptual framework of SCT, this study employed open-ended questions for assessment. Participants were presented with a thought-provoking, real-world problem scenario and asked to complete a response encompassing problem identification, hypothesis construction, and experimental verification. The design of such creative problem-solving tasks adhered to two key principles: first, the scenario must be related to curriculum standards to ensure the relevance of the assessment to students’ science learning experiences; second, the scenario itself must not be the central focus of any instructional unit to prevent the creativity measurement from devolving into a knowledge test (Enger and Yager, 1998).

Guided by these principles, this study designed three core open-ended task scenarios to elicit and assess the key cognitive processes of SCT. All selected scenarios were drawn from everyday life and closely linked to fundamental scientific principles, aiming to ensure ecological validity while avoiding direct testing of specific scientific knowledge.

Scenario 1, “Candle Combustion,” involves a widely recognized phenomenon encompassing multiple scientific concepts such as combustion conditions, changes in states of matter, chemical reactions, and gas composition. It provides participants with an open-structured platform for multi-faceted exploration.

Scenario 2, “Apple Discoloration,” is based on the common phenomenon of enzymatic browning (the browning of a cut apple surface in air). The process implies a wealth of investigable scientific questions, effectively stimulating conjecture and testing regarding the underlying chemical and biological mechanisms.

Scenario 3, “Steamed Bun Fermentation,” concretizes microbial activity and chemical changes by presenting differences in dough state under varying fermentation conditions. It involves biological and chemical principles of the fermentation process, offering participants another authentic, relatable, and deeply investigable problem space.

For each scenario, participants sequentially completed three continuous cognitive tasks: (1) Problem Identification: based on the presented scenario, list all scientific questions of interest, then independently select the one deemed most valuable for research as the core for subsequent thinking; (2) Hypothesis Construction: for the chosen problem, propose as many possible explanations as possible and formulate testable hypotheses; (3) Experimental Verification: select one of the proposed hypotheses and design a specific, operational experimental plan to verify its plausibility. Given the study’s focus on measuring the cognitive process of SCT and the practical constraints of large-scale testing, the “Experimental Verification” section focused on the conception and design of the experimental plan, not involving subsequent steps such as hands-on operation, data collection, or analysis.

### 4.2 Scoring rubric

The scoring for this study was based on the fundamental principle of the Consensual Assessment Technique, whereby experts familiar with a specific domain can make independent and reliable subjective judgments about the novelty and appropriateness of creative products based on their internalized creativity standards (Amabile, 1982). However, because assessing “appropriateness” in scientific contexts involves more complex domain-specific judgments, this study introduced a structured rater training component on top of the classic technique. The focus of the training was to establish a unified understanding among raters regarding the operational definitions of core constructs like “research value” and “essence,” rather than prescribing specific answers. This approach aimed to maintain judgments grounded in professional implicit consensus while enhancing inter-rater consistency, thereby achieving a balance between ecological validity and measurement reliability.

During the process of frequency calculation and scoring, responses that were semantically equivalent (i.e., expressed in different wording but referring to the same scientific question) were consolidated by the raters. Specifically, two raters first independently conducted preliminary coding for each response, then reached a consensus through discussion to identify responses addressing the same question in the following cases: (1) responses using different sentence structures but conveying the same core inquiry (e.g., “Why does the apple turn yellow?” and “What is the reason for the apple turning yellow?”); (2) responses posing detailed follow-up questions about the same phenomenon that essentially target the same underlying mechanism (e.g., “Why does it start turning yellow from the middle?” and “Whether the discoloration begins at the center?”). In cases of disagreement, a third subject matter expert was invited to provide arbitration, ensuring the accuracy of frequency statistics and the consistency of scoring.

### 4.3 Pilot testing and scale revision

This stage involved a systematic examination of the initial version of the SCTS through a small-scale pilot test. The purpose was to evaluate item quality, scoring procedures, and preliminary validity, leading to subsequent revisions.

#### 4.3.1 Pilot sample and procedure

The pilot testing involved 43 tenth-grade students from a key high school in a city in Shandong Province. Following the test, retrospective interviews were conducted with a subset of students. The interviews employed a “think-aloud” method, requiring students to verbally report their thought processes while completing each task scenario, including: how they understood the scenario description, how they identified problems, the basis on which they formulated hypotheses, and how they conceived experimental designs. The interviews were audio-recorded and subsequently transcribed. Two researchers independently analyzed the transcripts to confirm the inherent alignment between students’ responses and the constructs the scale intended to measure. Additionally, item analysis was conducted on the pilot data to examine the scale’s comprehensibility, feasibility, and item quality.

#### 4.3.2 Pilot results and revision

##### 4.3.2.1 Face validity and item quality

The think-aloud protocols provided evidence for the scale’s face validity from the perspective of students’ interpretation and cognition of the task scenarios. Interview results indicated that all students accurately understood the scientific content and task requirements of the three scenarios, with no misinterpretation or ambiguity regarding the instructions. During the problem identification stage, students’ thinking primarily revolved around the potential mechanisms underlying the observed phenomena. In the hypothesis construction and experimental verification stages, students were able to reason based on their existing scientific knowledge, and their thinking pathways were largely consistent with the three-stage theoretical model. Taken together, these findings suggest that the instrument possesses strong face validity as perceived by the target population. Additionally, 98% of students completed all three scenarios within 45 min, indicating that the time allocation for the test was reasonable.

Item analysis indicated the items demonstrated sufficient variability (standard deviations ranging from 0.89 to 1.21), and all item-total correlations reached moderate to high levels (*r* = 0.42–0.68), suggesting good item discrimination.

##### 4.3.2.2 Content validity

To examine the scale’s content validity, four experts (two university professors of science education and two experienced secondary school science teachers) were invited to evaluate it, employing a combination of quantitative and qualitative methods.

For the quantitative assessment, experts used a 5-point Likert scale (1 = “Very inadequate” ~ 5 = “Very adequate”) to rate scenario appropriateness (i.e., whether the three scenarios could effectively measure SCT) and task structure validity (i.e., whether the three-tiered “Problem Identification—Hypothesis Construction—Experimental Verification” structure accurately reflected the target construct). All dimension scores averaged above 4.0, indicating experts deemed the scale to have good content representativeness.

Fleiss’ Kappa coefficient was further used to assess inter-expert rating consistency. Following the criteria proposed by Zuehlke et al. (2009), the Fleiss’ Kappa coefficient in this study was 0.798, falling within the 0.6–0.8 range, indicating a high level of agreement among experts (see Table 1).

**Table 1:** Fleiss’ kappa test results for expert ratings.

Fleiss’ *κ*	*SE*	*z*	*p*	95% *CI*
0.798	0.17	4.79	< 0.001	[0.74, 0.85]

The qualitative assessment involved collecting experts’ specific suggestions for improving the clarity of item descriptions and structural rationality through open-ended questions, providing crucial guidance for subsequent revision.

##### 4.3.2.3 Inter-rater reliability check

To examine the operability of the scoring criteria, two trained raters (both with backgrounds in science education) independently scored 30 pilot responses. Results showed intraclass correlation coefficients (ICCs) of 0.87 for the Problem Identification dimension, 0.83 for Hypothesis Construction, and 0.85 for Experimental Verification. This indicates the scoring criteria were clear and feasible, with good inter-rater reliability.

##### 4.3.2.4 Scale revision

Integrating feedback from student pilot testing and optimization suggestions from expert review, the initial scale was revised. Revisions primarily focused on optimizing the instructions and item descriptions, and the scoring guide was updated accordingly, culminating in the final SCTS (Supplementary Appendix 1) and its revised scoring rubric (see Table 2).

**Table 2:** Scoring rubric for the scientific creative thinking scale (revised version).

Task	Score	Behavioral description
Problem identification	3	Proposes ≥2 unique questions demonstrating research value.
	2	Proposes 1 unique question demonstrating research value.
	1	Proposes a question with either uniqueness or research value (but not both).
	0	Questions lack both uniqueness and research value.
Hypothesis construction	3	Generates ≥2 substantive hypotheses that are empirically testable.
	2	Generates 1 substantive hypothesis that is empirically testable.
	1	Hypothesis is either substantive or testable (but not both).
	0	Hypotheses are neither substantive nor testable.
Experimental verification	3	Designs ≥2 elaborate and valid verification plans.
	2	Designs 1 elaborate and valid verification plan.
	1	Plan is valid but not elaborate, or elaborate but invalid.
	0	Plans are neither valid nor elaborate.

Operational definitions: (1) Unique questions are those generated by fewer than 10% of participants. (2) Valuable questions exhibit thoughtfulness or criticality and are amenable to further investigation. (3) Essential hypotheses address the core mechanism of the problem, independent of their factual correctness. (4) Testable hypotheses are those verifiable through specific empirical procedures. (5) Valid plans are designs capable of logically and rigorously testing the target hypothesis. (6) Elaborated plans are specific and operable, featuring clear steps, controlled variables, and concrete procedures.

### 4.4 Formal testing

#### 4.4.1 Formal sample and procedure

This study employed a two-stage sampling design to conduct exploratory factor analysis (EFA) and confirmatory factor analysis (CFA) of the scale, respectively. The EFA sample consisted of tenth-grade students recruited from three provinces in China: Zhejiang (eastern China), Shandong (northern China), and Jilin (northeastern China). A total of 189 scales were distributed, with 186 returned. After excluding four invalid responses, the final valid sample comprised 182 participants (valid response rate: 96%). Participants’ ages ranged from 16 to 17 years, including 94 females (51.6%) and 88 males (48.4%). The CFA sample was recruited separately from the same grade level (tenth grade) in Shandong Province. A total of 230 students participated, yielding 219 valid questionnaires (valid response rate: 95%). The age distribution of this sample was consistent with the EFA sample (16–17 years), comprising 112 females (51.1%) and 107 males (48.9%).

The scale was administered in a paper-and-pencil format in group settings to avoid systematic biases potentially associated with online surveys (Wright, 2005). The administration was conducted during regular class hours with permission from the schools and relevant teachers. Classroom science teachers or homeroom teachers supervised the sessions to ensure a consistent testing environment. Each student independently completed the scale, which contained three scenario-based tasks. The time allotted for each task was 15 min, resulting in a total completion time of 45 min. To mitigate social desirability bias, students were explicitly informed that there were no “correct answers” for the tasks, that all responses would be kept strictly confidential and used solely for academic research purposes, and that their individual answers would not be disclosed. Although class identifiers were included for administrative purposes, students were encouraged to respond based on their genuine thoughts and to work independently.

#### 4.4.2 Scoring procedure

To ensure scoring consistency, the two raters received systematic training on the revised scoring guide prior to formal scoring. Subsequently, 30 responses (approximately 16% of the sample) were randomly selected for independent pilot scoring. Following this, a consensus meeting was held, focusing specifically on responses where scoring discrepancies were ≥1 point. Raters articulated their reasoning for their scores, and consensus was reached through discussion, interpretation, and clarification of the scoring criteria. This iterative “independent pilot scoring–consensus meeting” process was repeated, each time using a new random sample of no fewer than 20 responses, until the intraclass correlation coefficient [ICC(2,1)] calculated for two consecutive iterations was ≥0.70, indicating an acceptable level of inter-rater agreement had been achieved. Subsequently, the raters independently performed the formal scoring for all 401 valid responses based on the finalized scoring rules. To illustrate the scoring criteria and enhance transparency, Appendix 2 presents annotated response exemplars for the Apple Discoloration scenario, including verbatim student responses and the rationale for each assigned score.

#### 4.4.3 Data analysis

To comprehensively evaluate the psychometric properties of the scale, data analyses were conducted from the perspectives of reliability and validity. Reliability analyses included: assessing the internal consistency of the total scale and each scenario-specific subscale using Cronbach’s *α* coefficient and McDonald’s *ω* coefficient; and evaluating the scoring consistency of two raters across the three core dimensions using the intraclass correlation coefficient (ICC). Validity analyses included: examining the underlying factor structure of the scale through Exploratory Factor Analysis (EFA)—employing Principal Axis Factoring (PAF) for factor extraction, utilizing Promax oblique rotation, and supplementing with parallel analysis to determine the number of factors to retain; further testing the robustness and model fit of this structure using Confirmatory Factor Analysis (CFA) with an independent sample; and examining criterion-related validity by analyzing Spearman’s rank correlations between the total scale score, scenario-specific scores, and students’ science academic achievement. Data analyses were conducted using SPSS 25.0 and AMOS 26.0 software.

## 5 Results

### 5.1 Descriptive statistics and item quality

Descriptive statistical analyses were conducted on the test data. Each item was scored on a 4-point scale ranging from 0 to 3, yielding a theoretical total score range of 0–27 for the SCTS. In the present sample, students’ actual scores ranged from 3 to 25, with a mean score of 13.45 (SD = 5.38), and the score distribution approximated normality. Item means ranged from 1.19 to 2.09 (out of a maximum of 3), with standard deviations ranging from 0.87 to 1.20. The proportions of participants scoring the minimum (0) or maximum (3) on any item were both below 10%, indicating no significant floor or ceiling effects and suggesting that the items possess good discriminatory power.

### 5.2 Reliability

#### 5.2.1 Internal consistency reliability

The SCTS demonstrated good internal consistency overall (Cronbach’s *α* = 0.87; McDonald’s *ω* = 0.89). In the Cronbach’s α analysis, item-total correlation coefficients for all items ranged from 0.42 to 0.68, indicating that each item effectively reflected the measurement objectives of the total scale. To verify the consistency of measurement across different scenario-based tasks, we calculated the reliability of the scenario-specific subscales using both Cronbach’s α and McDonald’s ω coefficients. As shown in Table 3, the α coefficients for the three scenario subscales ranged from 0.75 to 0.82, and the ω coefficients ranged from 0.76 to 0.85, all exceeding the acceptable threshold of 0.70. These results indicate that the scale possesses good internal consistency reliability.

**Table 3:** Reliability of scenario-specific subscales.

Scenario	α	ω	Items
Candle combustion	0.75	0.77	3
Apple discoloration	0.82	0.85	3
Steamed bun fermentation	0.75	0.76	3

α, Cronbach’s alpha coefficient; ω, McDonald’s omega coefficient.

#### 5.2.2 Inter-rater reliability

The intraclass correlation coefficient ICC (2,1) under a two-way random-effects model was calculated to assess inter-rater reliability. As shown in Table 4, the ICC values for the three dimensions ranged from 0.95 to 0.97, all exceeding the “good” threshold of 0.75 (Cicchetti, 1994), indicating excellent inter-rater agreement and that the scoring criteria were clear and reliably implemented.

**Table 4:** Intraclass correlations for inter-rater reliability by dimension.

Dimension	*ICC*	95% *CI*	*p*
Problem identification	0.95	[0.91, 0.97]	<0.001
Hypothesis construction	0.95	[0.93, 0.97]	<0.001
Experimental verification	0.97	[0.95, 0.98]	<0.001

ICC, intraclass correlation coefficient; Model: absolute-agreement, two-way random-effects (ICC[2,1]).

### 5.3 Validity

#### 5.3.1 Construct validity

Exploratory Factor Analysis (EFA). Exploratory factor analysis was conducted using the first subsample (*n* = 182). The suitability of the data for factor analysis was examined, yielding a Kaiser-Meyer-Olkin (KMO) measure of sampling adequacy of 0.796 and a significant Bartlett’s test of sphericity (χ^2^ = 1095.26, df = 36, *p* < 0.001), indicating that the data were appropriate for factor analysis. Principal Axis Factoring (PAF) was employed for factor extraction. Given the theoretical expectation of associations among the three dimensions, Promax oblique rotation was used to allow for correlations among factors (Montag and Walla, 2016). To determine the number of factors to retain, multiple criteria were employed. The Kaiser-Guttman criterion indicated that the first three factors had eigenvalues greater than 1.0; the scree plot revealed a distinct inflection point after the third factor, after which the trend leveled off; additionally, Parallel Analysis was conducted as a more robust standard (Hayton et al., 2004). Based on 1,000 randomly generated datasets, the observed eigenvalues were compared against the 95th percentile eigenvalues from the random data. The results showed that the eigenvalues for the first three factors exceeded their corresponding random counterparts, while the eigenvalue for the fourth factor fell below the random criterion, thus providing stable support for a three-factor structure. The three extracted factors cumulatively accounted for 70.189% of the total variance (see Table 5).

**Table 5:** Total variance explained by principal axis factoring.

Factor	Initial eigenvalues			Extraction sums of squared loadings			Rotation sums of squared loadings
	Total	% of variance	Cumulative %	Total	% of variance	Cumulative %	Total
1	3.920	43.556	43.556	3.124	34.711	34.711	2.735
2	1.315	14.611	58.167	1.874	20.822	55.533	2.388
3	1.082	12.022	70.189	1.319	14.656	70.189	1.194

Extraction Method: Principal Axis Factoring. Rotation Method: Promax with Kaiser Normalization. Parallel analysis (1,000 iterations, 95th percentile) confirmed the retention of three factors. Rotation converged in 5 iterations.

The rotated factor pattern matrix (see Table 6) revealed that all nine items demonstrated factor loadings above 0.78 on their corresponding primary factors, with no significant cross-loadings, indicating a clear factor structure. The three factors corresponded, respectively, to the theoretical model’s dimensions: “Problem Identification” (Items 1, 4, and 7), “Hypothesis Construction” (Items 2, 5, and 8), and “Experimental Verification” (Items 3, 6, and 9).

**Table 6:** Rotated factor pattern matrix from Promax rotation.

Item	Factor 1	Factor 2	Factor 3
Q1	0.898		
Q4	0.847		
Q7	0.885		
Q2		0.807	
Q5		0.812	
Q8		0.785	
Q3			0.915
Q6			0.906
Q9			0.887

Extraction Method: Principal Axis Factoring. Rotation Method: Promax with Kaiser Normalization.

The correlations among factors ranged from 0.462 to 0.523 (see Table 7), indicating moderate to strong positive relationships among the three factors. These intercorrelations support the use of oblique rotation and suggest that, while the dimensions are empirically distinct, they represent related aspects of a broader construct (Mostafa et al., 2025). The strongest correlation was observed between Problem Identification and Hypothesis Construction (*r* = 0.523), suggesting that these two dimensions may be mutually reinforcing. Experimental Verification showed slightly lower correlations with the other two factors (r ranging from 0.462 to 0.487), indicating a greater degree of distinctiveness for this dimension.

**Table 7:** Factor correlation matrix from Promax rotation.

Factor	Problem identification	Hypothesis construction	Experimental verification
Problem identification	1		
Hypothesis construction	0.523**	1	
Experimental verification	0.487**	0.462**	1

**p < 0.01.

Confirmatory Factor Analysis (CFA). Confirmatory factor analysis was conducted using the second independent subsample (*n* = 219). Based on the theoretical framework, the nine observed indicators (Q1–Q9) were specified to load onto three latent variables: Problem Identification, Hypothesis Construction, and Experimental Verification. The model fit results are presented in Table 8. The goodness-of-fit indices indicated that the three-factor model fit the data well: χ^2^/df = 3.009, which is below the recommended threshold of 5; CFI = 0.948 and TLI = 0.922, both exceeding the 0.90 criterion; RMSEA = 0.076, falling below the acceptable level of 0.08; and SRMR = 0.032, well below the 0.80 threshold. All fit indices met acceptable standards, demonstrating that the three-factor model exhibited good fit with the data.

**Table 8:** Fit indices for the CFA.

Fit indices	χ^2^	df	χ^2^/df	GFI	AGFI	CFI	TLI	RMSEA	SRMR
Value	72.227	24	3.009	0.910	0.832	0.948	0.922	0.076	0.032
Criteria of fit indices			<3 is excellent; 3–5 is good	>0.90	>0.90	>0.90	>0.90	<0.05 is excellent; 0.05–0.08 is acceptable	<0.05

#### 5.3.2 Convergent validity and discriminant validity

Composite reliability (CR) and average variance extracted (AVE) were calculated based on factor loadings. The results showed that CR values for all dimensions exceeded the recommended threshold of 0.70 (Raykov, 1997): Problem Identification CR = 0.9035, Hypothesis Construction CR = 0.8622, Experimental Verification CR = 0.8605. AVE values all exceeded the 0.50 threshold (Nunnally and Bernstein, 1994): Problem Identification AVE = 0.7575, Hypothesis Construction AVE = 0.6776, Experimental Verification AVE = 0.673. These results indicate that the scale possesses good convergent validity.

Discriminant validity concerns the extent to which items measuring different constructs are relatively uncorrelated (Hu and Bi, 2025). The square root of AVE for each factor (Problem Identification √0.7575 = 0.870; Hypothesis Construction √0.6776 = 0.823; Experimental Verification √0.673 = 0.820) exceeded its correlations with the other factors, satisfying the Fornell-Larcker criterion. This indicates that the scale demonstrates good discriminant validity.

#### 5.3.3 Criterion validity

Students’ science academic achievement was employed as an external criterion to examine its Spearman’s rank correlation with the total SCTS score. Given that the three task scenarios in the scale were all grounded in chemical principles, students’ scores on a standardized school-administered chemistry examination from the most recent end-of-semester assessment were selected as the criterion measure. This examination was conducted approximately 4 weeks prior to the SCTS administration and was not specifically designed for this study. The tests were developed by experienced chemistry teachers and reviewed by subject matter experts. To account for differences in examination difficulty across schools, raw scores were converted to within-school z-scores, thereby rendering all students’ scores comparable on a common scale. As shown in Table 9, the total scale score demonstrated a significant positive correlation with the transformed achievement scores (*ρ* = 0.55, *p* < 0.001). Scores on each scenario-specific subscale also showed significant, moderate positive correlations with achievement scores (ρ ranging from 0.50 to 0.57, all *p* < 0.001), providing preliminary evidence for the criterion-related validity of the scale.

**Table 9:** Correlation between academic achievement and scientific creative thinking performance.

Measure	ρ	*p*
Candle combustion	0.50	<0.001
Apple discoloration	0.57	<0.001
Steamed bun fermentation	0.55	<0.001
Total scale	0.55	<0.001

Spearman’s ρ reported. All *p*-values are two-tailed.

## 6 Discussion, limitations, and future directions

Guided by a three-stage cognitive model and integrating explicit and implicit assessment approaches, this study developed and initially validated the SCTS. The scale operationalizes SCT as a sequential process involving problem identification, hypothesis construction, and experimental verification. Empirical evidence indicates that the SCTS possesses sound psychometric properties, offering a novel assessment pathway and an empirical foundation for understanding the cognitive architecture of scientific creativity.

In terms of reliability, the SCTS demonstrated excellent properties. The internal consistency reliability of the total scale was good (*α* = 0.87, *ω* = 0.89), and the reliabilities of each scenario-specific subscale also reached acceptable levels (α = 0.75–0.82, ω = 0.76–0.85). These results indicate that the items reliably measure the latent construct of “scientific creative thinking.” Crucially, through systematic rater training and consensus-building procedures, exceptionally high inter-rater agreement was achieved for all three core cognitive dimensions (ICC ≥ 0.95). This outcome holds significant methodological importance: it suggests that by designing domain-specific, structured tasks combined with a standardized implicit judgment process, the objectivity and reliability of subjective scoring for complex cognitive constructs can be effectively enhanced. This addresses the longstanding challenges faced by traditional divergent thinking tests regarding predictive and ecological validity (Zeng et al., 2011). This study also provides a reference model for measuring domain-embedded higher-order thinking skills, one that balances ecological validity with psychometric rigor.

Regarding construct validity, exploratory factor analysis employing Principal Axis Factoring (PAF) with Promax oblique rotation, supplemented by parallel analysis to determine the number of factors to retain, robustly supported a three-factor structure, accounting for 70.189% of the total variance. All items demonstrated factor loadings exceeding 0.78 on their corresponding factors, indicating a clear factor structure that aligned precisely with the theoretical framework. Confirmatory factor analysis conducted with an independent sample further confirmed the adequate fit of the three-factor model (CFI = 0.948, TLI = 0.922, RMSEA = 0.076, SRMR = 0.032). Inter-factor correlations ranged from 0.46 to 0.52, reflecting moderate associations and indicating that the SCTS successfully operationalizes SCT as a multidimensional structure comprising interrelated yet distinct dimensions. These findings provide empirical support for the cognitive theory that conceptualizes scientific discovery as a systematic search across hypothesis and experiment spaces (Klahr and Dunbar, 1988), thereby offering psychometric evidence for the perspective that scientific creativity represents a domain-specific, structured cognitive process.

Regarding convergent and discriminant validity, the composite reliability (CR) for each dimension exceeded 0.86, and the average variance extracted (AVE) for each dimension exceeded 0.67, indicating that the scale possesses good convergent validity. The square root of the AVE for each factor (ranging from 0.82 to 0.87) was greater than its correlations with the other factors, satisfying the Fornell-Larcker criterion and further confirming the discriminant validity of the scale.

For criterion-related validity, the total SCTS score showed a moderate positive correlation with students’ academic achievement in science. This aligns with the theoretical expectation that domain knowledge provides a foundation for creative thinking (Anwar and Aness, 2012), offering preliminary evidence for the scale’s criterion validity.

It should be noted that the assessment in this study was conducted sequentially across the three stages of problem identification, hypothesis construction, and experimental verification, and that students’ performance may exhibit systematic differences across these stages. This variability does not represent measurement error but rather reflects the stage-specific characteristics of the SCT process. The cognitive tasks at each stage differ in nature: problem identification emphasizes observation and questioning, hypothesis construction relies on association and reasoning, and experimental verification stresses logical and operational design. Individuals possess different strengths across these cognitive components, reflecting the heterogeneous structure of scientific creativity (Hu and Adey, 2002). Furthermore, the degree of openness and difficulty varies across stages; the problem identification stage imposes a higher cognitive load, whereas the hypothesis and verification stages involve relatively more structured tasks (Lin and Lien, 2013). Therefore, this study treats the scores from the three stages as independent indicators reflecting distinct sub-constructs and does not advocate for simple summation or cross-stage comparisons. Future research could employ cognitive interviewing, eye-tracking, or other methods to further investigate differences in cognitive strategies across stages, or conduct task difficulty equalization to more precisely differentiate stage effects from individual differences.

The core theoretical contribution of this study lies in proposing an integrative assessment framework. The SCTS is not merely a transplantation of general creativity indicators into the science domain. Instead, it attempts a deep integration of explicit indicators of cognitive products with implicit judgments of domain appropriateness. Traditional divergent thinking tests primarily assess the novelty of products (e.g., fluency, originality), while the assessment of “appropriateness” or “effectiveness” is often superficial. Building on Sternberg’s implicit theory, this study recognizes that judgments of “appropriateness” in scientific creation are highly dependent on internalized domain norms. Therefore, it adopted and refined the Consensual Assessment Technique, allowing raters to provide comprehensive judgments on aspects like “research value,” “essence,” and “effectiveness” based on their implicit professional consensus. This methodological integration enables the SCTS to capture both the novelty and appropriateness dimensions of creativity simultaneously, offering conceptual and methodological reference for measuring domain-specific creativity that requires balancing “breakthrough” with “compliance.” Furthermore, by anchoring abstract scientific creativity in the specific cognitive sequence of “problem identification—hypothesis construction—experimental verification,” it provides an operational, testable theoretical model, facilitating future empirical research on related cognitive mechanisms.

Several limitations of this study should be acknowledged. First, the sample was limited to Chinese secondary school students; thus, the cross-cultural and cross-age generalizability of the findings requires further validation. Second, while the study examined internal consistency and inter-rater reliability, evidence for cross-time stability (test–retest reliability) was not assessed. Third, due to sample size constraints and the lack of sufficiently large and balanced subgroups, we were unable to test for measurement invariance across key demographic groups such as gender and school type. This limits our ability to confirm that the SCTS measures the same constructs in the same way across these populations. Fourth, although the significant correlation between SCTS scores and science academic achievement (*ρ* = 0.55) provides preliminary evidence for criterion-related validity, this finding should be interpreted with caution, as prior academic achievement and cognitive ability were not controlled for as potential confounding variables. Fifth, while the three task scenarios were designed to approximate real-life situations, they may still carry certain cultural specificities (e.g., the steamed bun fermentation scenario may be less familiar in some cultural contexts).

Future research should address these limitations in several ways. First, the psychometric properties of the SCTS should be further examined in more diverse and representative samples, employing longitudinal designs to assess cross-time stability and utilizing stratified sampling to conduct multi-group confirmatory factor analyses for testing measurement invariance. Second, future studies should include control variables such as prior academic achievement and cognitive ability to examine the incremental validity of the SCTS. Additionally, developing more culturally adaptable scenario banks or tasks targeting different scientific sub-domains, along with employing multiple criterion measures and multi-method approaches, will help to further enrich the validity evidence for the scale.

Looking ahead, the SCTS can serve as a foundational tool for exploring a range of deeper questions. For example, employing cognitive neuroscience methods to investigate the brain activity patterns corresponding to the three thinking stages and their dynamic interactions; examining the relationships between SCT and classic personality/cognitive variables such as openness, need for cognition, and executive functions; or using longitudinal studies to track its developmental trajectory and key influencing factors. Such work will not only deepen the understanding of SCT itself but also help integrate it more systematically into the broader landscape of individual differences and cognitive psychology.

## Data Availability

The datasets presented in this article are not readily available because the datasets generated and analyzed during the current study are not publicly available due to ethical and privacy restrictions. The primary data consist of written, open-ended responses from minor participants (secondary school students). Furthermore, the informed consent obtained from participants and their guardians did not include authorization for public data deposition. De-identified, aggregated data that underpin the findings reported in this manuscript are available from the corresponding author (Prof. Hualin Bi) upon reasonable request. Requests to access the datasets should be directed to Hualin Bi, bihualin@sdnu.edu.cn.
