# Supplementary material for: Measuring scientific creative thinking: development and validation of a process-oriented instrument
Source: Front Psychol. 2026 Mar 3;17:1762573. doi: 10.3389/fpsyg.2026.1762573 (PMC13040359; doi:10.3389/fpsyg.2026.1762573)
Supplement: Supplementary file 1 [file Supplementary_file_1.pdf]

Appendix 1: *Scientific Creative Thinking Scale*

Name: ______________Gender: ______________School: ______________Class:______________

Dear Student,

Thank you for participating! This is not a conventional exam but a series of image-based scenario activities assessing your abilities in:

·Problem Identification (identifying curiosity-driven questions),

·Hypothesis Construction (predicting possible causes), and

·Experimental Verification (designing plans to test your predictions).

There are no “correct” answers, and responses will remain confidential for academic research.

Please note: Each activity requires at least 15 minutes. New ideas may emerge while you reflect—feel free to add them to your responses.

Activity 1

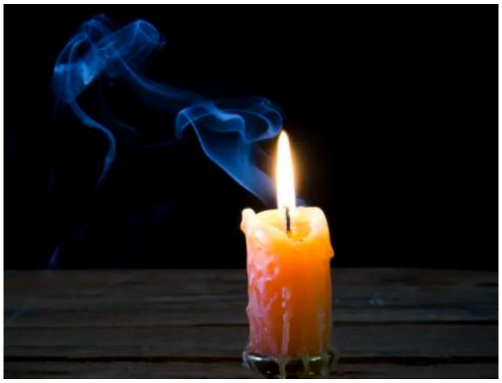

Activity 2

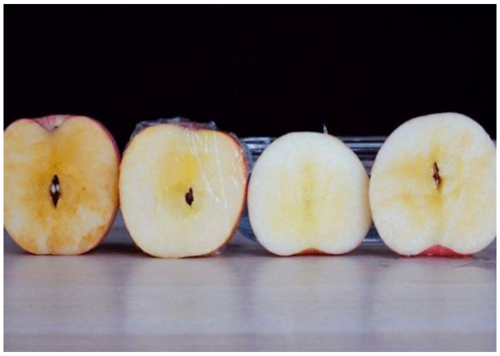

Activit 3

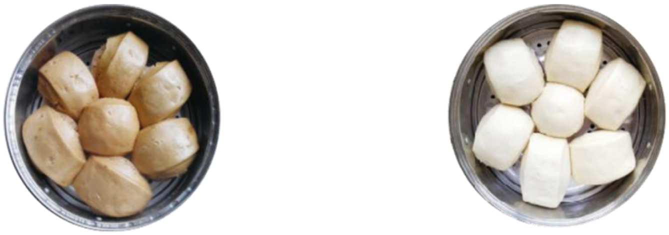

(a) When yeast fermentation fails, adding sodium bicarbonate results in steamed buns with a yellowish color and a slightly alkaline taste.

(b) Steamed buns produced by directly adding sodium bicarbonate and vinegar to the dough.

Core Activity Template (Applied identically to Activities 1, 2, and 3)

1. Problem Identification

·Observe the provided image carefully.

·Document all scientific questions you want answered (label numerically: Question 1, Question 2, ...).

·List all the questions generated.

2. Hypothesis Construction

·Select one focus question (e.g., *For Question 1*).

·Propose testable hypotheses (label numerically: Hypothesis 1, Hypothesis 2, ...).

·Generate multiple hypotheses for the chosen question.

3. Experimental Verification

·Choose one target hypothesis (e.g., *For Question 1, Hypothesis 2*).

·Design a detailed, implementable procedure for hypothesis verification (label: Plan 1, Plan 2).

·*Consider employing various approaches to verify this hypothesis.*
