# Supplementary material for: Measuring scientific creative thinking: development and validation of a process-oriented instrument
Source: Front Psychol. 2026 Mar 3;17:1762573. doi: 10.3389/fpsyg.2026.1762573 (PMC13040359; doi:10.3389/fpsyg.2026.1762573)
Supplement: Supplementary file 2 [file Supplementary_file_2.pdf]

Appendix 2: *Scoring Example*

This section uses Student Jia's responses to the "Apple Discoloration" scenario to illustrate the scoring process and rationale for each stage.

I. Problem Identification

Original Response:

"*Why do apples turn yellow, why use a black curtain, why do apples start turning yellow from the middle, and why wrap the apples in plastic wrap.*"

Scoring Process:

Student Jia posed four questions, analyzed as follows:

Question 1, "Why do apples turn yellow," has research value, but as 47% of the sample also posed this question, it does not meet the uniqueness criterion.

Question 2, "Why use a black curtain," was posed only by this student, thus meeting the uniqueness criterion. However, the question pertains to an irrelevant feature of the experimental setup and is not directly related to the phenomenon of apple discoloration, so it lacks research value.

Question 3, "Why do apples start turning yellow from the middle," arises from observation of the scenario and has research value. However, as 16% of the sample posed the same question, it does not meet the uniqueness threshold (<10%).

Question 4, "Why wrap the apples in plastic wrap," was posed by 12% of the sample, thus not meeting the uniqueness criterion. However, the question pertains to the role of the plastic wrap and has some investigative value.

Overall Assessment:

Student Jia did not pose a question that possessed both uniqueness and research value. According to the scoring criteria for the Problem Identification stage in Table 2, the score for this stage is 1 point.

II. Hypothesis Construction

Original Response:

"*The apple is oxidized upon contact with oxygen. The divalent iron ions (*Fe^{2+}*) in the apple are oxidized to trivalent iron ions (*Fe^{3+}*), which appear yellow.*"

Scoring Process:

Student Jia's hypothesis contains two core elements: (1) the discoloration results from an oxidation reaction; (2) the oxidized substance is Fe^{2+}, with Fe^{3+} as the oxidation product. This hypothesis addresses the core mechanism of the phenomenon and is empirically testable, demonstrating both fundamentality and testability.

Overall Assessment:

Student Jia proposed a hypothesis concerning the essence of the problem that is testable. According to the scoring criteria for the Hypothesis Construction stage in Table 2, the score for this stage is 2 points.

III. Experimental Verification

Original Response:

"*Prepare two identical apples, cut them in half. Leave one half exposed to the air, and seal the other half inside plastic wrap. Wait 5 minutes, then remove the apple and observe the discoloration. Extract juice from both apple halves into two test tubes. Add KSCN solution to each; if it turns red,* Fe^{2+} *is present. If there is no color change, add* Cl_2 *water; if it then turns red,* Fe^{2+} *is present.*"

Scoring Process:

Student Jia designed two sub-experiments: (1) A comparison between exposure to air and sealing in plastic wrap to test the hypothesis that contact with oxygen causes oxidation. (2) A chemical test using KSCN and chlorine water to test the hypothesis that Fe^{2+} is oxidized to Fe^{3+}. This design can effectively test the proposed hypotheses. The experimental variables are clear, the operational steps are detailed, and the material choices are appropriate, demonstrating both effectiveness and sophistication.

Overall Assessment:

Student Jia proposed an experimental design capable of testing the hypothesis that is complete, detailed, and operable. According to the scoring criteria for the Experimental Verification stage in Table 2, the score for this stage is 2 points.
